# Cross-species imputation and comparison of single-cell transcriptomic profiles

**DOI:** 10.1101/2023.10.19.563173

**Published:** 2023-10-20

**Authors:** Ran Zhang, Mu Yang, Jacob Schreiber, Diana R. O’Day, James M. A. Turner, Jay Shendure, Christine M. Disteche, Xinxian Deng, William Stafford Noble

**Affiliations:** 1 Department of Genome Sciences, University of Washington; 2 eScience Institute, University of Washington; 3 Department of Biomedical Informatics and Medical Education, University of Washington; 4 Department of Genetics, Stanford University; 5 Brotman Baty Institute for Precision Medicine, University of Washington; 6 Sex Chromosome Biology Laboratory, The Francis Crick Institute; 7 Howard Hughes Medical Institute; 8 Allen Center for Cell Lineage Tracing; 9 Department of Laboratory Medicine and Pathology, University of Washington; 10 Department of Medicine, University of Washington; 11 Paul G. Allen School of Computer Science and Engineering, University of Washington

## Abstract

Cross-species comparison and prediction of gene expression profiles are important to understand regulatory changes during evolution and to transfer knowledge learned from model organisms to humans. Single-cell RNA-seq (scRNA-seq) profiles enable us to capture gene expression profiles with respect to variations among individual cells; however, cross-species comparison of scRNA-seq profiles is challenging because of data sparsity, batch effects, and the lack of one-to-one cell matching across species. Moreover, single-cell profiles are challenging to obtain in certain biological contexts, limiting the scope of hypothesis generation. Here we developed Icebear, a neural network framework that decomposes single-cell measurements into factors representing cell identity, species, and batch factors. Icebear enables accurate prediction of single-cell gene expression profiles across species, thereby providing high-resolution cell type and disease profiles in under-characterized contexts. Icebear also facilitates direct cross-species comparison of single-cell expression profiles for conserved genes that are located on the X chromosome in eutherian mammals but on autosomes in chicken. This comparison, for the first time, revealed evolutionary and diverse adaptations of X-chromosome upregulation in mammals.

## 1 Introduction

The magnitude of a gene’s expression may vary across species, and this variation may contribute to or be representative of morphological or trait evolution [1, 2]. Thus, comparing gene expression profiles across species has the potential to offer valuable insights into a wide range of questions related to, for example, which genes have adapted to new regulatory machineries and functions during evolution, and how gene expression changes when a gene moves to a different chromosomal context or when a gene’s copy numbers varies across organisms (e.g., genes located on two sets of autosomes in chicken and on the single X chromosome in male mouse). Furthermore, understanding transcriptional differences between model organisms and humans will greatly enhance our ability to transfer insights gained from model organism studies into a human context.

Previous studies have compared transcriptional differences among organisms based on bulk and single-cell gene expression measurements. However, comparison of bulk gene expression profiles across species [3–6] may not fully capture cellular heterogeneity and may suffer from imbalanced cell type composition of tissues across species [7]. Recently, single-cell profiles have been widely generated to capture cell-specific expression profiles and mitigate the issue of uncaptured cell heterogeneity in bulk samples, but direct transcriptional comparisons are difficult because of the challenge of matching cells across species. Moreover, single cell measurements suffer from sparsity, noise, variable sequencing depth, and potential batch effects. As a result of these limitations, current studies instead perform cross-species matching or comparison at the cell type level [8–12]. Unfortunately, this approach requires accurate cell type calling and matching across species and, similar to bulk comparisons, fails to take into consideration single-cell variability.

More importantly, although single cell data are widely available for certain model organisms, such as mouse, single-cell expression atlases in human and non-model-organisms are still far from complete, due to limitations of sample availability and accessibility (e.g., it is difficult to retrieve human brain samples, as well as fetal or pediatric tissues, especially in disease conditions). Because of these limitations, studies of transcriptional evolutionary patterns are restricted to tissues or cell types in species that have existing, high-quality measurements.

To address these challenges, new computational methods are needed that can (1) predict gene expression profiles for missing cell types and biological contexts and (2) directly compare expression profiles across species at single-cell resolution, without relying on external cell type annotations. Although several deep learning-based methods enable cross-species prediction of perturbation effects from high-throughput screens, these methods rely on discrete cell type labels and are not designed to predict and compare cellular gene expression in wildtype physiological conditions across species [7, 13, 14]. We hypothesize that a neural network model that can decompose single cell profiles into species factors and cell factors invariant of species will allow us to make single-cell, cross-species prediction and comparison by swapping the species factor corresponding to each cell.

**Figure 1:**
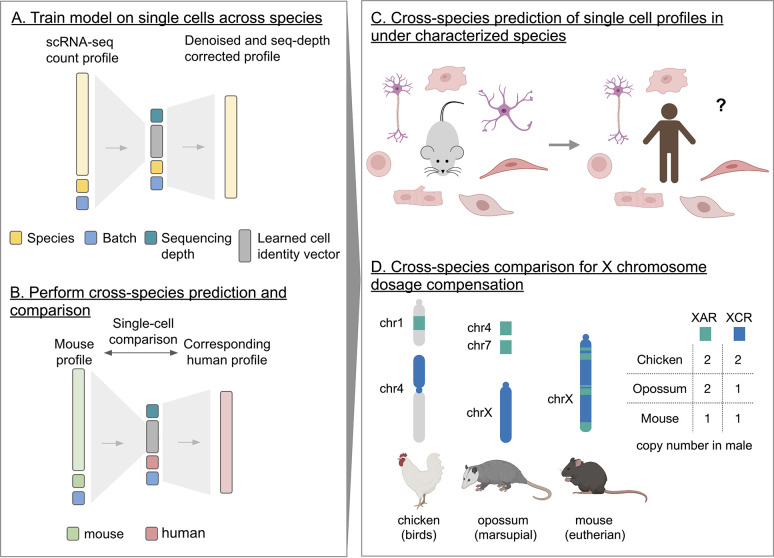
Icebear’s cross-species prediction and comparison framework. (A) Icebear is trained on single-cell RNA-seq profiles across species. Specifically, Icebear uses conditional variational autoencoders that predict cellular profiles from a combination of species and batch factors, as well as species-invariant cell factors. (B) Once the model is trained, we can swap the species factor to predict a (e.g., mouse) cell’s corresponding profile in another species (e.g., human). (C) Icebear can be used to predict single-cell profiles in species (e.g. human) where the corresponding cell type has not been profiled. (D) Icebear can also perform cross-species comparison at the single-cell level, revealing X chromosome up-regulation patterns during evolution.

We are motivated in part by the study of sex chromosome evolution, which would benefit from methods for single-cell comparison and prediction of gene expression across species. In mammals, males have a single X chromosome and a gene-poor Y chromosome, whereas females have two X chromosomes. X chromosome upregulation (XCU) has been proposed to evolve in response to gene loss due to Y-chromosome degeneration during sex chromosome evolution [15–20]. XCU increases expression of many X-linked genes to balance gene expression between the single X chromosome and two sets of autosomes in diploid male XY cells. In female XX cells, X inactivation silences one of the two X chromosomes in females to avoid hyperactivation. Evolutionary studies have shown that the X chromosome of eutherian mammals has arisen from ancestral autosomes in two major steps: first, an autosome gave rise to the so-called X conserved region (XCR) in the ancestor of both eutherian and metatherian mammals [18]. Today, the XCR is represented by the whole X chromosome of marsupials (e.g., opossum) and corresponds to about two thirds of the eutherian X chromosome. The latter acquired the so-called X added region (XAR) by translocation of autosomal pieces to the XCR, which resulted in the larger conserved X chromosome of eutherian mammals (e.g. human, mouse, rat). Chromosomes that demonstrate homology to the XCR and XAR can be identified in marsupials (X chromosome and parts of autosomes 4 and 7) and in birds (parts of autosomes 1 and 4 in chicken) (Figure 1D).

While XCU has been clearly demonstrated in Drosophila and C. elegans, the mechanisms of XCU in mammals are still debated [21–25]. Importantly, which X-linked genes are upregulated and at which levels at each evolutionary step is still unclear. This is largely because most studies have relied on transcriptomic approaches to compare expression between groups of X-linked and autosomal genes. However, direct expression comparisons of evolutionarily conserved genes before and after becoming X-linked are limited or impeded by different data normalization methods, and by the lack of appropriate samples to directly compare species across tissues and cell types [4, 5].

Here we propose a deep learning model, Icebear, that induces a non-sparse version of single-cell expression data and performs cross-species prediction and comparison at single-cell resolution. Using several publicly available datasets, we demonstrate that Icebear is able to integrate single-cell expression profiles across species, batch and tissue types, and predict single-cell profiles in missing cell types across species. In addition, we show that, on the basis of mouse data, Icebear can accurately predict transcriptomic alterations in human Alzheimer’s disease (AD) versus control samples, thereby enabling the transfer of knowledge from single-cell profiles in mouse disease models to human. After several cross-species validation experiments based on public datasets, we applied Icebear to predict and compare gene expression changes across eutherian mammals (mouse), metatherian mammals (opossum) and birds (chicken), using our in-house generated sci-RNA-seq profiles with minimal cross-species batch effects. By doing so, Icebear reveals gene expression pattern shifts across species that support the existence of mammalian XCU and suggest the extent and molecular mechanisms of XCU vary among mammalian species and among X-linked genes with distinct evolutionary origins.

## 2 Methods

### 2.1 Multi-species single cell profile generation

Mixed-species scRNA-seq data were generated by a three-level single-cell combinatorial indexing approach (sci-RNA-seq3) [26]. Adult brain and heart from both male mouse and chicken were purchased from BioChemed Services, and male opossum adult brain was provided by J. Turner (MRC, UK). The data were collected by indexing cells from each species by reverse transcriptase barcoding and then processing them jointly, in which case the species identity of each cell was known based on the sequence barcode.

### 2.2 Assigning species labels to cells and mapping reads

Mapping reads from sci-RNA-seq3 experiments to profile hundreds of thousands of single cells from multiple species’ samples is complicated by the possibility of cells from different species entering the same doublet during sample preparation and three-round combinatorial cell barcoding. Accordingly, our pipeline begins by mapping each read to multiple species and retaining only reads that map uniquely to a single species. This step allows us to detect and remove species-doublet cells with reads from more than one species.

The detailed protocol is as follows:

1. For a given sample, create a multi-species reference genome by concatenating the reference genomes of all the species used in that sample.
2. Map all of the reads to the multi-species reference, retaining only reads that map uniquely. We used the STAR aligner [27] with the following parameters: --outSAMtype BAM Unsorted --outSAMmultNmax 1 --outSAMstrandField intronMotif --outFilterMultimapNmax 1.
3. Remove PCR duplicates.
4. Eliminate any read that maps to an unassembled scaffold, mitochondrial DNA, or any locus that is marked as a repeat element by RepeatMasker (http://www.repeatmasker.orgi). The repeat elements by RepeatMasker were retrieved from UCSC genome browser [28], with the exception of opossum, where we ran RepeatMasker to generate them. Repeat elements were removed using BEDtools [29].
5. For each cell, count the total number of remaining reads that map to each of the three species.
6. If the sum of the second- and third-largest counts is greater than 20% of all counts, then mark the cell as a species-doublet and eliminate it.
7. Label the remaining cells according to their generating species.

Having identified the species origin for each cell, we then re-map the reads associated with each single-species cell only to its corresponding species, retaining only the reads that map uniquely within that species. The parameters used for this step were --outSAMtype BAM Unsorted --outSAMmultNmax 1 --outSAMstrandField intronMotif.

The re-mapping is run using the pipeline developed by the Brotman Baty Institute (BBI) (https://github.com/bbi-lab/bbi-sci/). The first two steps of mapping multi-species reference were modified from their pipeline. The reference genomes and annotations were from the BBI, using assembly GRCm38 for Mus musculus, ASM229v1 for Monodelphis domestica, and GRCg6a for Gallus gallus (Ensembl release 99) [30]. The genes were also filtered to match the ones used by BBI.

### 2.3 Reconciling orthology relationships

To simplify the model and focus on the most straight-forward cross-species transcriptional changes, we would like to establish one-to-one orthology relationships among genes in the various species included in our study. The Biomart resource [31, 32] at Ensembl reports orthology relationships between genes in a many-to-many fashion, each with an associated percent identity score. To reduce this data resource to a one-to-one mapping, our approach takes into account the following considerations.

- We use the percent identity score to resolve ambiguities. If, for example, two mouse genes are mapped to two opossum genes with four edges, then we select the two edges that create a one-to-one mapping with maximal score.
- In some cases, transitive relationships can be used to fill in missing edges. For example, if gene A in human has an ortholog B in mouse, B has an ortholog C in chicken, but A has no ortholog in chicken, then we can add an edge from A to C.

Formally, we represent our problem using an undirected graph G in which vertices V are genes and edges E represent orthology relationships. Each vertex v∈V has an associated species label s(v)∈{human,mouse,rat,opossum,chicken}. Our goal is to eliminate edges from this graph so as to ensure that every node is connected to at most one node in each of the other species This is done by building a graph $G^{\prime }=\left\{V^{\prime },E^{\prime }\right\}$ such that $V^{\prime }\subset V$, as follows. First, we reconcile mouse with each other species by creating a one-to-one mapping between genes in the two species. We do this in a greedy fashion for two species A and B by ranking all edges connecting A and B (i.e., all “high confidence” edges in Biomart) in decreasing order by percent identity and then adding an edge to $G^{\prime }$ if and only if neither of its corresponding vertices already has an associated accepted edge. Second, we fill in transitive relationships. This is done by creating a one-to-one mapping between genes, using the same greedy algorithm as before, but for each pair of non-mouse species. We then search for “transitivity” triangles (A,B,C) that fulfill the following criteria:

- s(A)= human
- s(B)≠ human and s(C)≠ human
-
s(B)≠s(C)

- The edge from A to B is in $G^{\prime }$.
- $G^{\prime }$ does not contain any edge connected to C.
- The one-to-one mapping between s(B) and s(C) connects B to C.

In this case, we add to $G^{\prime }$ the edge connecting A to C. This transitivity step is done iteratively over the non-mouse species in order of evolutionary distance; i.e., the species B and C are considered in the following order: (mouse, opossum), (mouse, chicken), (opossum, chicken). For each of the resulting triples of species, transitivity triangles are selected in a greedy fashion by sorting the triangles in decreasing order by the percent identity associated with the edge connecting B to C.

At the end of this process, in the graph $G^{\prime }$ each mouse vertex has at most one connected neighbor in each of the non-mouse species. Note that we make no attempt to ensure that the pattern of orthology relationships respects the species tree. Thus, in principle a mouse gene might have an ortholog in chicken but none in opossum. This is possible in the case of a gene deletion event along the opossum lineage. This process yielded 10,030 genes with orthologs shared across the three species, 70 of which were added through transitive relationships.

This process yielded 10,030 genes with orthologs shared across the three species, 70 of which were added through transitive relationships.

### 2.4 Data preprocessing

#### 2.4.1 In-house multi-species dataset

To perform cross-species prediction and comparison, we performed the following preprocessing steps:

1. Retain genes that have orthologs shared across all species.
2. Remove all mitochondrial reads.
3. Remove cells with <200 UMIs.
4. Remove genes expressed in fewer than 50 cells across all the datasets.

This process resulted in a gene (n=9878) by cell (m=561340) matrix (Table 1), the median UMI is 450.

#### 2.4.2 Public datasets

Gene expression profiles were downloaded from a multi-species primary motor cortex (M1) dataset [33], human and mouse cell atlases [34, 35] and an Alzheimer’s disease study [36].

For the M1 dataset, we used the expert-curated cell type annotations in each dataset, which contains 10 major cell type annotations that are further subdivided into 45 high-resolution cell type annotations. We only focused on cell types that are annotated as homologous between human and mouse based on expert curation. After applying the above gene filtering, mapping and cell filtering steps, 233,296 cells and 13,924 genes are retained for the downstream analysis.

For the human and mouse atlases, we trained Icebear on cells collected in the adult stage, which contains 288,886 cells and 12,367 genes. For the purpose of evaluation, cell types were determined based on expert annotations drawn from the original paper, and we only made predictions on cell types with ≥ 25 cells in both human and mouse. To make sure our predictions are not confounded by batch or donor effects, we only evaluated on the subset of cell types that exist in tissues that do not exhibit large donor effects in humans. To do that, we calculated Euclidean distances based on normalized pseudobulk profiles across all human adult tissue samples, and we retrieved tissues in which samples from different donors are closest to each other.

For the Alzheimer’s disease dataset, we retrieved single cells from human and 7-month-old mice. For the purpose of evaluation, we mapped cell type annotations from mouse to human based on the cell clusters called in the original paper, and we only validated on cell types with one-to-one mappings across species. No additional filtering was performed. This dataset was merged with the M1 dataset (with the same ortholog mapping approach above) to increase the cell numbers for model training. 440,689 cells and 12,474 genes are retained in the joint dataset.

Because the model takes raw counts as input, no further data normalization is needed.

### 2.5 Icebear cross-species prediction model

Our model reconstructs each scRNA-seq profile using three sets of factors representing, respectively, cell identity, species and batch. These factors are concatenated and fed into a neural network that reconstructs the original gene expression profile. The model is adapted from a conditional variational autoencoder framework [10]. Species, tissue and batch factors are encoded using one-hot encoding, and cell factors are learned as n-dimensional vectors, where n is a hyperparameter to be tuned.

Intuitively, each gene expression count (X) in a specific cell is estimated based on three learned variables: the sequencing depth-corrected mean $\left(\mu^{\prime }\right)$, dispersion of the negative binomial distribution (r), and logit of the dropout event (p). The reconstruction loss consists of the log likelihood of the raw count x and sequencing depth-corrected estimation using a zero-inflated negative binomial (ZINB) loss:

(1) $${loss}_{\text{reconstr}}=-zinb\cdot \mathrm{loglik}\left(X,\mathrm{ZINB}\left(d*\mu^{\prime },r,p|X,b,s,t\right)\right),$$

where d is the sequencing depth. The model also regularizes the cell embeddings by applying a KL divergence loss between the posterior distribution (Q) of cell embeddings (z) given batch (b), species (s) and tissue (t) factors and the prior distribution (P, standard multivariate normal distribution):

(2) $${loss}_{KL}=D_{KL}\left[Q\left(z|X,b,s,t\right)∥P\left(z,b,s,t\right)\right]$$

Thus, in each epoch we minimize the the following loss:

(3) $${loss}_{cVAE}={\text{loss}}_{reconstr}+{loss}_{KL}$$

To further guide the alignment of cells across species, optionally, we adopted the idea of generative adversarial networks (GANs) [37], where we used a discriminator to distinguish cells from different species, and then we trained the model to fool the discriminator and learn cell embeddings invariant to species. Specifically, we divided the model training into two major iterative steps. In the first step, we trained a single-layer discriminator to predict species labels (s) based on cell embeddings, to distinguish species of origin from learned cell embeddings. In this step, the model aims to minimize the discriminator loss calculating the discrepancy between true species label (s) and predicted label (D(z)):

(4) $${loss}_{dis}=CE(D(z|X,b,s,t),s)$$

In the second step, we fixed the parameters in the discriminator and tried to optimize for reconstruction and fooling the discriminator by minimizing ${loss}_{cVAE}-{loss}_{dis}$. The above steps are iterated per training epoch. This GAN option is provided as an option in the hyperparameter search.

In each prediction task, we held out all cells in a cell type or tissue in the target species as the test set, to mimic the actual use case where we would like to predict cellular profiles in an unseen context. This set of cells was not seen by the model during training and was used to evaluate prediction performance. For the rest of the cells, because our model does not rely on cell type annotations, we randomly assigned 10% to the validation set (with a cap of 20,000 cells), and the rest of cells were used as the training set. Each model was trained until the validation losscVAE stopped decreasing for 45 consecutive epochs. We then selected the model with minimum validation loss.

We selected model hyperparameters using a grid search strategy. We considered two hyperparameters: a Boolean indicating whether to include the discriminator layer or not, and the dimension of the bottleneck layer of 25, 50, 100. The number of hidden layers in the encoder and decoder are fixed to 2. Among these possible hyperparameter settings, we selected the model that yielded the largest LISI score [38] across cell embeddings learned from different species on the validation set.

To perform cross-species prediction on single cells, we first learned cell embeddings from cellular profiles in the known species and then concatenated the cell embeddings with the species factors of the target species to make cross-species predictions through the trained decoder architecture. This process produced sequencing depth-normalized and denoised single cell gene expression in the target species for each corresponding cell in the source species.

### 2.6 Cross-species prediction evaluation metric

To evaluate the prediction performance, we made use of expert-curated cell type labels and cell type matching across species, and we asked whether the model can accurately predict pseudobulk gene expression profiles of the missing cell types across species.

For each cell type, we held out its profile in the target species and made predictions using the corresponding cell type’s profile in the source species. Pearson correlation between the true and predicted pseudobulk profiles was used to assess the prediction performance (“prediction”). Pseudobulk profiles were calculated as the average of sequencing-depth-normalized gene expression profiles across cells, to put the true and predicted profile on the same scale.

We compared the prediction performance with three baselines.

1. Donor baseline (“donor_baseline”): a “cheating” baseline that calculates the mean similarity across donors within the held-out test set.
2. Species baseline (“species_baseline”): similarity between the true profile and the corresponding cell type’s profile in mouse, with genes transferred to the target species through ortholog mapping.
3. Celltype baseline (“celltype_baseline”): we first identify the cell type that has the most similar pseudobulk gene expression profile to the held out cell type in the source species and then calculate the similarity between its corresponding cell type in the target species and the true profile.

To make a fair comparison with the performance of the donor baseline, we also calculated the mean of similarity measurements between our predictions with each donor in the test set (“indiv_predictions”).

To measure the prediction error with regard to the original gene expression magnitude and variation, for each held out cell type, we calculated the relative prediction error based on pseudobulk gene expression values as $\frac{|{\text{human}}_{\text{pred}}-{\text{human}}_{\text{ori}}|}{|{\text{mouse}}_{\text{ori}}-{\text{human}}_{\text{ori}}|}$.

### 2.7 Evaluation of Alzheimer’s disease profile prediction

To evaluate how well we can predict human AD profiles based on mouse models, we tried to place ourselves in the actual use cases when studying human diseases, which is to find differentially expressed genes and the direction and magnitude of expression alterations between AD and wild type (WT) in human samples. Because the predicted expression values in Icebear are denoised continuous values, it is not straightforward to statistically compare Icebear’s predictions with the sparse, count-based measurements in the original single-cell profiles. Therefore, we evaluated Icebear’s performance based solely on the direction and magnitude of gene expression changes in predicted AD vs WT profiles in human. These patterns are compared against the observed gene expression alternation patterns between the original AD versus WT profiles in human, based on Pearson correlation. Because studies of mouse disease models usually directly use the gene expression alterations in mouse as a proxy to understand human disease (when human samples are unavailable), we included gene alteration patterns in AD versus WT mouse as a baseline prediction, and calculated its correlation with gene expression alterations in human.

To capture both the direction and magnitude of gene expression alterations in AD and WT, we used the log2 fold change (log2FC) based on pseudobulk profiles (normalized to library size of 10,000) as the gene expression alteration measurement. To further avoid large, noisy log2FCs from genes with low expression values, we added 1 to all pseudobulk gene expression profiles before calculating log2FC.

### 2.8 Cross-species X-linked gene expression changes during evolution

To assess the pattern of XCU during mammalian sex-chromosome evolution, we first trained the model on all datasets and then compared gene expression changes between each pair of species by directly swapping the species factors. To do that, we used the trained Icebear model to retrieve cell embeddings based on single-cell profiles in mouse, and we predicted each cell’s corresponding gene expression profiles in the target species by appending the cell embedding with the target species factors. This approach allowed us to produce a denoised prediction of the gene expression profile of a given cell in the same or a different species. To make sure that the expression values are comparable across species, we normalized each cell’s gene expression profile based on 2,401 housekeeping genes in mouse [39]. Then we compared gene expression changes across species and, for each gene, we calculated the log2 fold change (log2FC) between the two species for each cell, and we took the median value across all cells as its overall log2 fold change. Genes were then grouped into three categories based on their evolutionary origin:

1. Genes located in the X-added region (XAR) genes were added to the X chromosome in eutherian mammals, i.e. these genes are X-linked in eutherian mammals (e.g., mouse) but are autosomal in metatherian mammals (on chromosomes 4/7) (opossum) and appear on chromosome 1 in chicken.
2. Genes located in the X-conserved region (XCR) are X-linked in both eutherian and metatherian mammals, but are autosomal (on chromosome 4) in chicken.
3. Genes located on autosomes in mammals and chicken.

To test whether genes in the XAR or XCR tend to become upregulated when becoming X-linked (i.e. lose one copy in males), we performed a one-sided, one-sample Wilcoxon signed-rank test with the null hypothesis that the median log2FC within that gene group is smaller than or equal to −1. To test whether XCU is stronger in mouse than opossum, we performed a one-sided, one-sample Wilcoxon signed-rank test with the null hypothesis that the median log2FC (between mouse and opossum) within that gene group is greater than or equal to 0. Similarly, to test whether the dosage of gene expression is fully compensated (i.e. each X-linked gene is upregulated by two-fold to achieve the same expression level that it had when it was autosomal), we performed a one-sided, one-sample Wilcoxon signed-rank test with the null hypothesis that the median log2FC (between mouse and chicken) within that gene group is greater than or equal to 0.

## 3 Results

### 3.1 Icebear accurately predicts cell type profiles across species

Icebear is a deep learning model that is designed to integrate cross-species, single-cell profiles (Figure 1A, Methods 2.5). Once trained, the model decomposes each observed cellular gene expression measurement into several components, corresponding to cell, batch and species. Prior to applying the model in a prospective fashion, we carried out several validation experiments to verify that the model works as intended.

**Figure 2:**
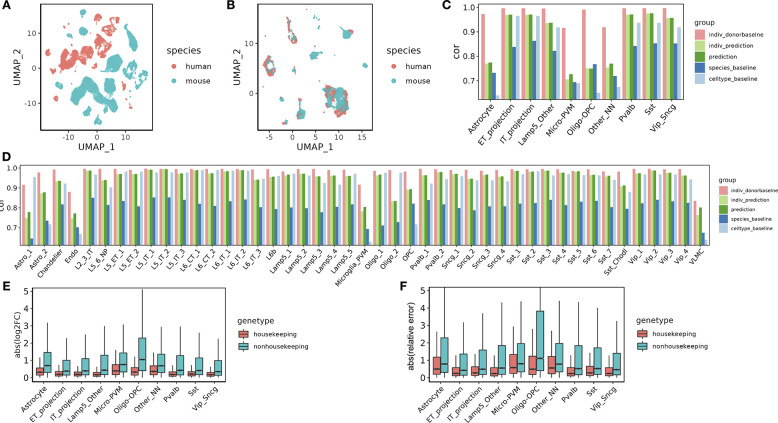
Alignment and prediction of single-cell profiles from mouse to human in primary motor cortex. (A) UMAP of cells across species, colored by species. (B) UMAP of cell embeddings learned in Icebear, where cell embeddings are independent of species factor. (C) Barplot showing Pearson correlation coefficient (cor) between predicted and observed gene expression profiles in missing major cell types in human motor cortex. For each cell type, the Pearson correlation coefficient is compared between Icebear’s prediction (dark green bar) and two baselines (species and cell-type). We also compared “indiv_donorbaseline” (correlation between individual donors) with Icebear’s correlation with individual donors (“indiv_prediction”, light green bar). (D) Similar to C, we evaluated Icebear’s performance on predicting high-resolution cell type specific profiles. (E) Boxplots of genes absolute log2FC between predicted and true human profiles per cell type (grouped by whether they are housekeeping genes or not). (F) Boxplots of genes relative prediction error across cell types.

First, we hypothesized that we could reduce or eliminate effects associated with species or batch factors by manipulating the model appropriately. To test this hypothesis, we trained the model on a public dataset derived from multiple species, and we assessed whether the model could eliminate the effect of species in the learned cell factors. Specifically, we used as input a dataset with primary motor cortex (M1) cells from both human and mouse [33], and we mapped genes across species via orthology [40]. The original dataset shows clear separation by species in the context of 2D visualization via UMAP (Figure 2A). We then investigated whether Icebear could be used to remove this species-specific effect. Accordingly, we plotted a UMAP visualization of the learned cell embeddings (Figure 2B). This visualization shows minimal segregation of cells by species, suggesting that Icebear can correct species-specific effects and align single cells across species.

The primary goal of Icebear is to jointly model gene expression data from human and mouse using factors representing species, gene, and cell identity, thereby enabling cross-species prediction and comparison. Ideally, the model could then be used to answer questions such as, “What would the expression profile of this mouse cell look like if it were instead a human cell?” Unfortunately, validating the accuracy of such a predictor is impossible. We therefore adopted an alternative validation approach.

To validate Icebear’s cross-species prediction performance, we made cross-species predictions at the level of individual cells but evaluated the predictive accuracy at the level of cell types. For this analysis, we used the cell type annotations produced in the original M1 study [33]. To mimic the real life scenario where we have uncharacterized biological contexts, we trained Icebear using a dataset in which one cell type in human was held out entirely, and we then used the trained model to predict gene expression profiles from the same cell type in mouse. Aggregating these predicted single-cell expression profiles yields a predicted pseudobulk profile that can then be compared, via Pearson correlation, to the pseudobulk profile of the held-out cells.

Before carrying out this experiment, we designed three “baseline” predictors to provide comparators for our model (Methods, Section 2.6). The first baseline (“donor baseline”) provides a “cheating baseline” on the predictive accuracy: we directly compute as our performance measure the mean Pearson correlation between pseudobulk profiles for the test cell type across all donors in the held-out test set. This baseline is cheating, in the sense that it has access to the data in the test set; however, the idea is that the empirical variance of the performance of this baseline on data from different individuals within a species provides a rough upper bound on how well any predictor could possibly perform on this task. The second baseline (“species baseline”) predicts that gene expression values do not change at all between species; thus, for a given cell type, the predicted human gene expression profile is equal to the corresponding mouse profile, with genes transferred from mouse to human through ortholog mapping. The third baseline (“cell type baseline”) is stronger than the species baseline, because it requires that we have access to cell type annotations in both species. In that setting, the cell type baseline makes a prediction for the held-out cell type by (1) finding the cell type in the source species whose pseudobulk profile most closely resembles that of the held-out cell type, and (2) identifying the corresponding cell type in the target species. The prediction for the held-out cell type is then the pseudobulk profile drawn from its neighboring cell type. Note that this baseline is somewhat unfair to Icebear, which is not given access to cell type labels in either species.

Validating on the cell types annotated in the M1 dataset, we find that Icebear outperforms both the species and cell-type baselines. We first applied the validation protocol to the 10 general motor cortex cell types, observing that in 9 out of 10 cases, Icebear outperforms both baselines (Figure 2C). The one exception is Oligo-OPC in which our model only outperforms the cell-type but not species pipeline. To further evaluate how well our model can predict single-cell profiles with fine cell type resolution, we trained Icebear on the same dataset but held out and evaluated the prediction based on high-resolution cell type annotations (Figure 2D). In this setting, Icebear outperforms the species baseline in all 45 cell types and outperforms the cell-type baseline in 33 of the 45 cell types ($p=5.62\times 10^{-5}$), Wilcoxon one-sided signed rank tests). Note that our model tends to perform better in neurons than non-neuronal cells, especially endothelial cells, microglia-PVM (perivascular macrophage) and VLMC (vascular leptomeningeal cells). As expected, our method performs worse than the “donor baseline.” This suggests that though the model can perform general cross-species imputation, it may fail to capture some cell type-specific evolutionary effects.

Because Icebear performs non-linear projection of data across species, we hypothesize that a gene with large functional divergence during evolution and across cell types may not only follow a general cross-species translation algorithm, but may also participate in species- and cell-type-specific adaptation processes. Thus, we expect that the expression values of functionally diverged genes may be poorly predicted across species. To test this hypothesis, we investigated whether housekeeping genes, whose functions are more likely to be conserved across species, can be more accurately predicted compared to non-housekeeping genes. To do that, we calculated absolute log2FC between the predicted and observed gene expression values in human (Figure 2E). Indeed, of all major cell types tested, Icebear achieved better predictive power for housekeeping genes compared to non-housekeeping genes. To further control for potential bias caused by baseline expression and expression differences across species, and to investigate how much Icebear’s prediction improves upon the species baseline, we calculated the absolute value of the ratio between the prediction error (predicted human vs. original human) and cross-species difference (original mouse vs. original human) (“relative prediction error”, Methods 2.6, Figure 2F). Again, Icebear is able to correct for species-specific effects more accurately for housekeeping genes than non-housekeeping genes. These results point to a potential limitation to any cross-species model, since the cell-type specific evolutionary divergences, which are a product of evolutionary selective pressure, may contribute to the gap between the predicted and observed profiles.

### 3.2 Icebear can generalize across tissues and datasets

To test whether Icebear can generalize across tissues in another dataset, we trained the model on single-cell profiles from the human and mouse cell atlases [34, 35]. In this validation experiment, we focused on single-cell profiles collected in the adult stage (Methods 2.4.2). We held out each major cell type from human and trained Icebear to predict expression profiles based on the corresponding cell types in mouse. To ensure a fair comparison that considers tissue-specific variations, we evaluated the cell-type-specific profile prediction per tissue, even though such information is not used in our training.

**Figure 3:**
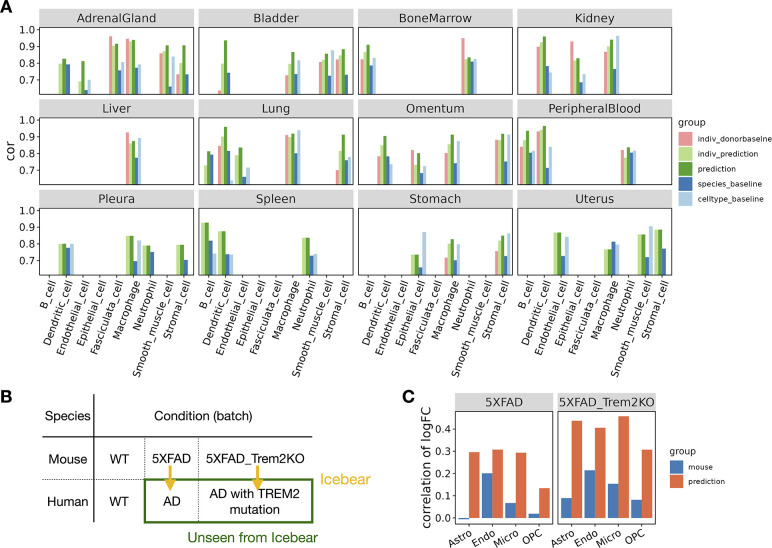
Prediction of human cell-type specific profiles across tissues and conditions. (A) Barplot showing Pearson correlation coefficient (cor) between predicted and true gene expression profiles in each held-out cell type per tissue. For each cell type, the Pearson correlation coefficient is compared between Icebear’s prediction (dark green bar) and the species and cell type baselines. We also compared “indiv_donorbaseline” (correlation between individual donors) with Icebear’s correlation with individual donors (“indiv_prediction”, light green bar). (B) Illustration of Icebear’s framework for predicting held-out human AD profiles from mouse AD models. (C) Comparison of predicted and true gene expression log2FC patterns in AD vs. WT. Barplots show Pearson correlation coefficients between observed and predicted log2FC in human (“prediction”, orange), and between original log2FC in human and mouse (“mouse”, blue).

The results of this experiment suggests that Icebear can outperform both the species-specific and cell-type-specific baseline in 33 cases out of 41 total (Figure 3A). Interestingly, Icebear outperforms the donor baseline in 19 out of 25 cases (when more than one donor exists for that tissue and cell type). These results suggest that Icebear provides a robust estimation of single-cell profiles across species and thus can be broadly applied to computationally impute single cell profiles in human based on measurements in mouse across tissues and cell types.

### 3.3 Icebear can transfer findings from a mouse Alzheimer’s disease model to human

As a final validation and demonstration of a key use case, we test the hypothesis that Icebear can predict gene expression alterations in disease conditions versus healthy controls in human, based on healthy human samples and disease models in mouse. To test this hypothesis, we trained Icebear on human and mouse single-cell profiles in primary motor cortex (used in the above section), control samples in both human and mouse from an AD study, as well as profiles in the AD mouse model (Figure 3B) [36]. Once the model was (which was not certified by peer review) is the author/funder, who has granted trained, we applied it to predict the held-out profiles in human AD samples (green rectangle, Figure 3B) and calculated the log2FC of predicted gene expression between AD and WT samples in human. To evaluate how well the predicted gene alteration pattern agrees with the true pattern, we compared it against the true log2FC pattern derived from the original human AD versus control samples, using Pearson correlation (Figure 3C, orange, Methods 2.7). To make sure Icebear captures more informative disease signatures than the mouse model itself, we compared the true log2FC pattern in human against the log2FC pattern in mouse AD versus WT samples per cell type (Figure 3, blue). In all uniquely mapped cell types, Icebear’s prediction outperforms the mouse baseline, suggesting that by projecting single-cell profiles from mouse to human, Icebear is able to retrieve more accurate gene alteration patterns in human, compared to the original mouse experiments.

### 3.4 Icebear reveals X chromosome upregulation patterns during evolution

**Figure 4:**
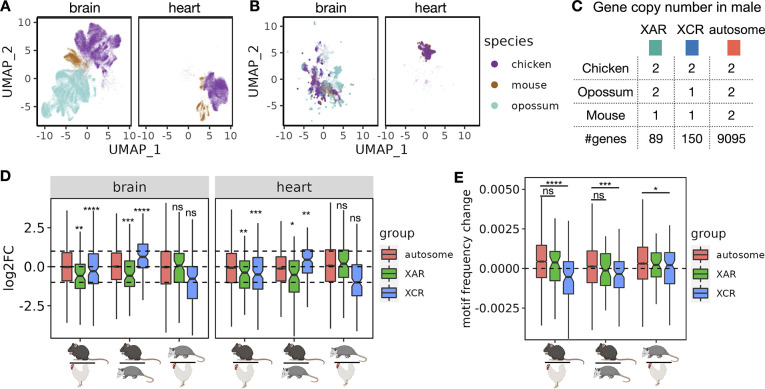
X chromosome gene upregulation pattern across species. **(A)** UMAP based on original input profiles. (B) UMAP based on cell embeddings learned from Icebear. (C) Gene copy number changes in males across species. (D) Boxplot of log2 fold change of genes across species, with genes grouped by their X-linked pattern. Statistical significance of XCU events are calculated using Wilcoxon one-sample rank sum test and subjected to multiple hypothesis correction. (E) Boxplot of GGACH motif frequency changes across species, grouped by the same rule as C. Statistical significance calculated on the hypothesis that genes in XAR and XCR are likely to have less m6A motif binding when moved from autosome to X chromosome, compared to genes in autosomal regions.

Having established the ability of Icebear to capture species-specific effects, we next apply the model prospectively, using it to investigate the pattern of expression change across species in specific classes of genes. Essentially, our model allows us to ask, for any given cell, how its expression pattern would change if that cell had been in a different species. We are particularly interested in asking whether gene expression increases from halved gene dose when autosomal genes become X-linked and only have a single copy in XY males during mammalian sex chromosome evolution (Figure 1D, Figure 4C). To do that, we collected single cell RNA-seq profiles in male chicken, opossum and mouse brain samples, as well as male chicken and mouse heart samples (Figure 4A, Methods 2.4.2, with opossum heart not measured). We then applied Icebear on the dataset and predicted each cell’s gene expression changes when we swap its species factor across chicken, opossum and mouse (Figure 4B, Methods 2.8). For each gene, we can do this analysis on a cell-by-cell basis, asking how its expression changes when the cell changes from, say, chicken to opossum or opossum to mouse.

We find that when genes lying on the X conserved region (XCR) changed from the chicken autosome 4 (chr4) to the opossum X chromosome, the median gene expression log2FC between opossum and chicken do not fall significantly above or below −1 $\left(p=1.7\times 10^{-1}\right.$ in brain and $p=1.3\times 10^{-1}$ in heart, Wilcoxon onesample rank sum test, Figure 4D). Interestingly, these XCR genes show significant up-regulation on mouse X chromosome as shown by the median gene expression log2FC between mouse and chicken $\left(p=3.0\times 10^{-7}\right.$ in brain and $p=4.8\times 10^{-4}$ in heart), suggesting that XCU of XCR genes is more prominent in eutherian mammals than in marsupials.

In addition, for genes at X added region (XAR) that changed from the chicken autosome 1 (chr1) to the X chromosome in eutherian mammals after the divergence from metatheria marsupials (e.g., opossum in which these genes on chr4 or chr7), we observed their median log2FC between mouse and chicken, and between mouse and opossum, are significantly more than −1, suggesting X upregulation also occurs for this group of genes in mouse (Figure 4D). Furthermore, there are no significant changes observed of XAR genes changing from chicken to opossum, when they are both on autosomes, which agrees with prior expectation ($p=6.1\times 10^{-1}$ in brain and $p=8.0\times 10^{-2}$ in heart). Comparing mouse with opossum or chicken, our finding also agrees with and further supports the general notion that X-upregulation does not usually fully compensate for the half-dosage effect [17], since XAR and XCR genes tend to have logFC less than 1 (in XCR regions when comparing mouse to opossum) or 0 (in other XCU comparisons) (adjusted $p\leq 0.5\times 10^{-2}$ for all cases). Notably, Icebear is able to predict XCU patterns in heart, where single-cell measurements in opossum are unavailable.

A recent study has indicated that transcripts from X-linked genes including XAR and XCR genes tend to have lower levels of m6A (and thus less GGACH motifs) than those from autosomal genes, which results in more stable X transcripts compared to autosomal transcripts and thus contribute to XCU [24]. With this evidence, we hypothesize that the changes in m6A levels when genes move across species are related with whether the gene is in the XAR/XCR or autosomal. To test this hypothesis, and provide an orthogonal validation to XCU event, we retrieved the GGACH motif frequency in coding sequence (CDS) regions from chicken, opossum, and mouse [24], and ask whether there is a decrease in motif frequency for XAR/XCR orthologs when moved from chicken autosomes to mouse X chromosomes, compared to those from autosomal orthologs (Figure 4E). Indeed, we found that XCR orthologs show a significant decrease in the motif frequency between mouse and opossum or between mouse and chicken, when compared to autosomal orthologs. This agrees with Icebear’s prediction where XCU in the XCR is most significant in mouse (Figure 4D). In support of this, we only found marginal significance of motif frequency differences for XCR orthologs between opossum and chicken, in comparison to autosomal orthologs $\left(p=4.1\times 10^{-2}\right)$. More intriguingly, we didn’t see significant changes in motif frequency for XAR orthologs compared to autosomal orthologs between mouse and chicken or between mouse and opossum, suggesting that XCU in the XAR may not be adapted through enhanced RNA stability via reduced m6A motifs in the CDS region.

## 4 Discussion

In this study, we proposed Icebear, a machine learning model for cross-species prediction and comparison of single-cell gene expression data. We demonstrate Icebear’s utility in predicting missing cell type-specific profiles between species, accurately transferring gene alterations identified in a mouse disease model to the corresponding human disease context, and identifying gene expression alterations during evolution in response to X chromosome dosage changes.

In our analysis, we observed that Icebear’s prediction accuracy varies by cell type. For example, the accuracy in predicting expression values of human non-neuronal cells on the M1 dataset tends to be lower than that of other cell types. There could be two reasons for this variability. First, previous studies have found that glia cells are more evolutionarily diverged [41], so a general cross-species algorithm trained on cell types other than astrocytes may not accurately capture astrocyte-specific variations. Cells with immune functions such as microglia may be especially variable among species due to different exposure to pathogens. The other possible reason is that neuronal cell types are more abundant and their profiles and functions are more cohesive than those of glial cell types, which could lead to an easier machine learning task. In line with this explanation, we observed that housekeeping genes to be more accurately predicted across species, presumably because housekeeping genes tend to be functionally conserved across species and cell types and thus are more easily captured by Icebear’s projection framework. In future, a closer look at the genes whose predicted expression profiles are very different from their observed profiles could potentially reveal genes that adapt to the species-specific environment in a cell-type specific manner.

Icebear also shows improved accuracy at recapitulating transcriptomic perturbation patterns (based on log fold changes) in human AD based on mouse disease models, compared to traditional disease studies that directly map gene perturbation patterns across species by orthologs. This finding suggests a limitation of disease knowledge transfer through ortholog mapping, and demonstrates the potential of applying Icebear to more accurately transfer single-cell level perturbations such as disease signatures and drug responses from model organisms to human. In the future, we plan to develop statistical methods to rigorously assess the significance of predicted gene expression perturbations and compare them with the perturbation pattern based on original gene expression profiles, by leveraging ideas proposed by Boyeau *et al*. [42].

Icebear reveals that there is an increase of expression levels of X-linked genes compared to their autosomal orthologs in mouse, supporting the hypothesis that XCU occurs during sex-chromosome evolution (Ohno’s hypothesis) [43]. The level of upregulation varies among individual X-linked genes, suggesting a gene-by-gene adjustment. Multiple transcriptional and post-transcriptional types of regulation have been suggested to explain XCU in mammals [21, 25, 44–47]. The most recently proposed regulatory mechanism is reduced GGACH motifs at X-linked versus autosomal transcripts, which results in X-specific reduced m6A levels and thus enhanced RNA stability [24]. Surprisingly, we found that XCR but not XAR genes show a significant decrease in frequency of GGACH motifs compared to autosomal genes in mouse, suggesting differences in XCU mechanisms dependent on the evolutionary origin of the XCR and XAR. Interestingly, X inactivation, which has been proposed to counteract XCU in females (reviewed in Disteche *et al*. [16]), is also more complete for genes in the XCR versus the XAR [48].

In the future, we envision Icebear to be a general tool to (1) augment the effort of measuring complete profiles of human cells, (2) predict gene transcriptional changes in under-characterized human contexts by leveraging mouse models, and (3) study the evolutionary changes of transcription regulation, as well as divergence of cell types and genes. Icebear can also be extended in a straightforward fashion to perform cross-species prediction and comparison of other data modalities (e.g. protein quantity, epigenetic marks), when there are shared feature spaces across species.

## Figures and Tables

**Table 1:** Cell counts in the final dataset.

species	tissue	batch1	batch2	batch3

mouse	brain	33569	—	—
opossum	brain	2664	235178	—
chicken	brain	8927	167873	—
mouse	heart	—	—	17403
chicken	heart	—	28003	67723

## Data Availability

We are working with the 4D Nucleome Consortium to make the sci-RNA-seq data publicly available.
