# Supplementary material for: Cross-species imputation and comparison of single-cell transcriptomic profiles
Source: bioRxiv. 2024 Aug 12:2023.10.19.563173. Originally published 2023 Oct 20. Preprint. [Version 2] doi: 10.1101/2023.10.19.563173 (PMC10614954; doi:10.1101/2023.10.19.563173)
Supplement: 1 [file NIHPP2023.10.19.563173V2-supplement-1.pdf]

bioRxiv preprint doi: https://doi.org/10.1101/2023.10.19.563173; this version posted August 12, 2024. The copyright holder for this preprint (which was not certified by peer review) is the author/funder, who has granted bioRxiv a license to display the preprint in perpetuity. It is made available under aCC-BY 4.0 International license.

Supplementary information

S1 Supplementary methods

S1.1 Comparison with SATURN

One of the key strengths of Icebear is its ability to predict and assess how a gene's orthologs change expression across species. In particular, by making use of one-to-one ortholog mapping, Icebear can directly swap species factors to measure gene expression changes for orthologous genes during evolution. In contrast, SATURN uses macrogenes to perform cross-species cell alignment but not expression changes, and by design is incapable of predicting gene expression changes across species [14].

To compare Icebear with SATURN, we therefore can only evaluate the two methods with respect to cross-species alignment. To do so, we trained SATURN and Icebear separately on the primary motor cortex (M1) dataset [35] and human and mouse cell atlases [36, 37], because these two datasets have matching cell-type annotations across species. For each method, we used the LISI score to measure, for each cell type, how well cells from different species are aligned. The SATURN paper adjusts a single hyperparameter, the number of macrogenes, so we experimented with values of 1000, 2000, 3000, and 4000. At 5000 macrogenes, SATURN exceeded the available GPU memory (8 GB).

S1.2 Comparison with Harmony+kNN

We repeat the same protocol and evaluation of cell type prediction in the motor cortex dataset, where we hold out a cell type in human one at a time, run Harmony [13] to align the rest of the cells across species, retrieve the k-nearest human cells of each mouse cell from the held-out cell type, and use the pseudobulk profiles of all the k-nearest human cells as the baseline prediction. We tuned twice as many hyperparameters (12, rather than 6) for this method, including the number of principal components (PCs) used for Harmony (n=25, 50, 100), and the number of neighboring cells used to make the prediction (k=5, 20, 80, 320).

To select the best hyperparameters, we first choose the number of PCs based on cross-species cell alignment (calculated by the LISI score). We then select the best k based on a "cheating" method, where we pretend we already know the held-out cell type's nearest neighboring cell type correspondingly in human and mouse, and we select the best model that gives the best pseudobulk Pearson correlation prediction on the neighboring cell type. This method creates an unfair baseline for Icebear, as Icebear does not leverage any cell type information during the training or model selection stages.

S1.3 Multi-species evaluation

To address the question of how Icebear works on more than three species and evolutionarily distant species, we constructed a five-species dataset, consisting of our in-house generated heart dataset collected from mouse and chicken, along with other public heart datasets from human [51]), frog (*Xenopus laevis*) [52] and zebrafish (*Danio rerio*) [53]. No shared batches exist across these four datasets, making it infeasible to perform cross-species comparison.

To test cross-species alignment and prediction, we first performed one-to-one orthology mapping following the protocol described in the manuscript (Methods 2.3). The frog dataset separately measures genes from the L (long) and S (short) homologous chromosome sets. To approximate one-to-one orthology mapping, we took the sum of gene expression counts across the two sets of chromosomes for each gene. Overall, 8675 ortholog groups were mapped across all five species. Meanwhile, without the transitive mapping step, only 7697 genes were included. We then retained orthologs with measurements across all five species and only retained the biggest batch from the human, frog, and zebrafish datasets. Next, we filtered out genes expressed in fewer than 50 cells and cells with fewer than 100 genes expressed. The above process ends up with 3064 genes and 36,227 cells.

Finally, we trained the Icebear model on this five-species dataset. In the first task, we used the default hyperparameters by setting the dimension of the embedding layer to 25 and ignoring the discriminator step, and then training Icebear on all cells. Using the resulting model, we asked whether cells from different species are aligned to the same space. As a control, we also generated cell embeddings derived from UMAP

bioRxiv preprint doi: https://doi.org/10.1101/2023.10.19.563173; this version posted August 12, 2024. The copyright holder for this preprint (which was not certified by peer review) is the author/funder, who has granted bioRxiv a license to display the preprint in perpetuity. It is made available under aCC-BY 4.0 International license.

on the original measurement. In the second task, we identified shared cell type labels from species where cell type annotation is available (i.e., human, frog, and zebrafish), held out each cell type in human, and trained an Icebear model on the rest of the cells. We report the Pearson correlation between the original held-out profile and Icebear-predicted human cell type-specific profile based on the corresponding cell type in the frog or zebrafish (Methods 2.6).

bioRxiv preprint doi: https://doi.org/10.1101/2023.10.19.563173; this version posted August 12, 2024. The copyright holder for this preprint (which was not certified by peer review) is the author/funder, who has granted bioRxiv a license to display the preprint in perpetuity. It is made available under aCC-BY 4.0 International license.

S2 Supplementary figures

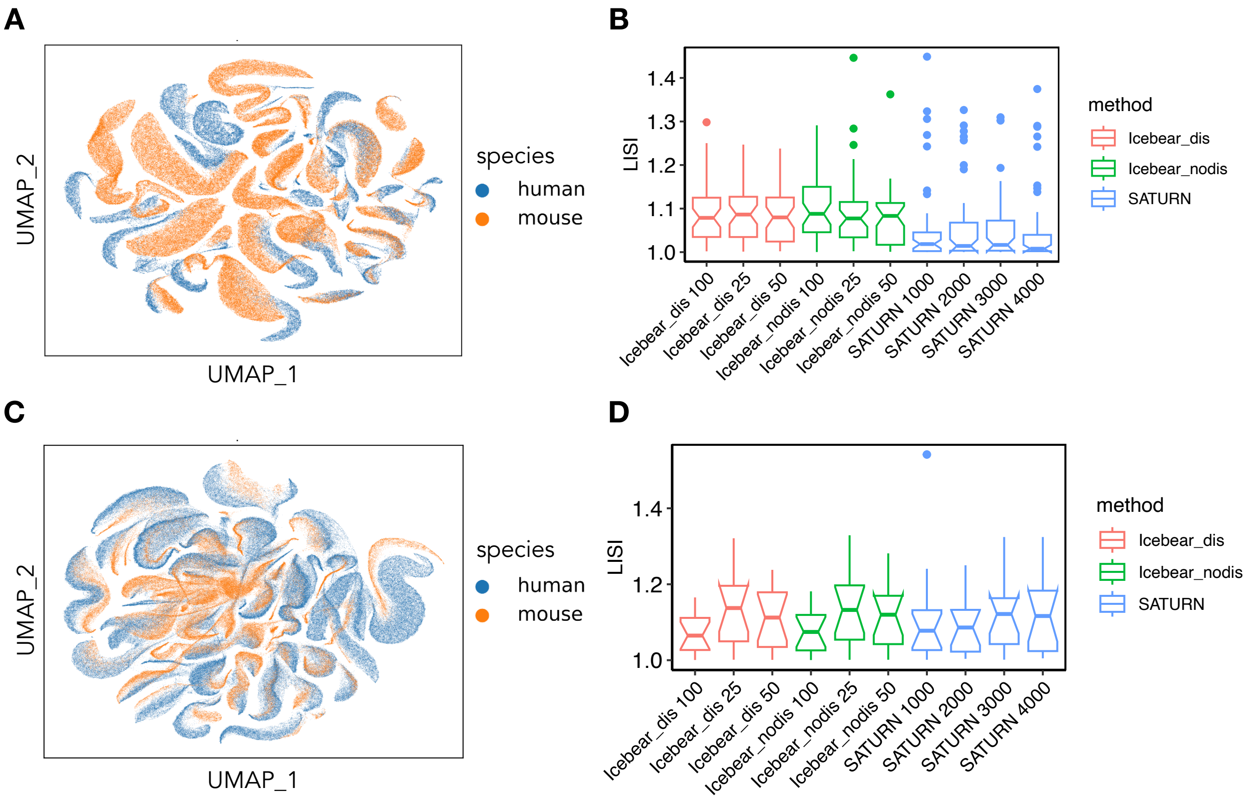

Figure S1: **Icebear is comparable to or more accurate than SATURN at aligning single cells across species.** (A) UMAP of SATURN-aligned cell embeddings of the motor cortex dataset, colored by the species each cell comes from. Median UMI across cells: mouse = 12060; human = 20056. (B) Boxplot distributions of LISI scores measuring how well mouse and human cells from the motor cortex dataset are aligned per cell type. All hyperparameters tuned are shown. (C) Similar to (A), UMAP of SATURN-aligned cell embeddings of the cell atlas dataset, colored by the species each cell comes from. Median UMI across cells: mouse = 688; human = 591. (D) Similar to (B), the LISI score distribution of cross-species alignment per cell type is measured on the cell atlas dataset.

bioRxiv preprint doi: https://doi.org/10.1101/2023.10.19.563173; this version posted August 12, 2024. The copyright holder for this preprint (which was not certified by peer review) is the author/funder, who has granted bioRxiv a license to display the preprint in perpetuity. It is made available under aCC-BY 4.0 International license.

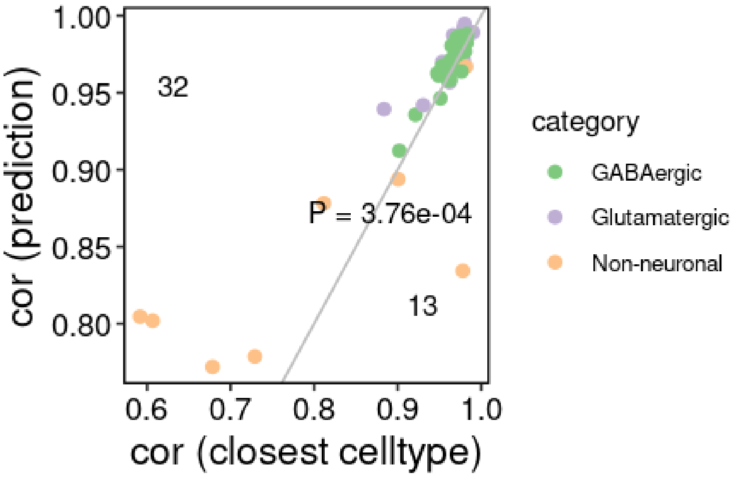

Figure S2: **Comparison of Icebear and Harmony+kNN baseline** Pseudobulk Pearson correlation between the predicted and original profile in the mouse motor cortex dataset. Each dot indicates a cell type, colored by the major cell type annotation. The x-axis represents results generated by the Harmony+kNN baseline, and the y-axis shows results generated by Icebear. Numbers indicate the number of cell types off-diagonal. A p-value is calculated using Wilcoxon one-sided signed rank tests.

bioRxiv preprint doi: https://doi.org/10.1101/2023.10.19.563173; this version posted August 12, 2024. The copyright holder for this preprint (which was not certified by peer review) is the author/funder, who has granted bioRxiv a license to display the preprint in perpetuity. It is made available under aCC-BY 4.0 International license.

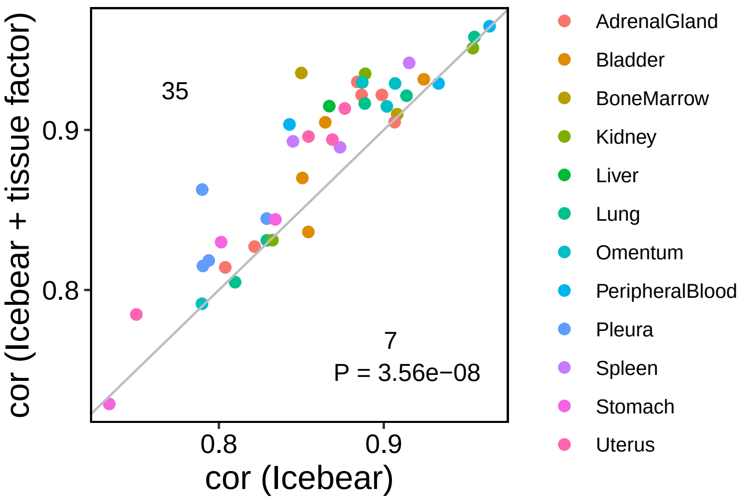

Figure S3: **Adding a tissue factor improves Icebear's performance.** Pseudobulk Pearson correlation between the predicted and original profile in the cell atlas dataset. Each dot indicates a cell type, colored by the tissue it is extracted from. The x-axis represents results are generated by the Icebear model without the tissue factor, and the y-axis shows results are generated by Icebear with a tissue factor. Numbers indicate the number of cell types above and below the diagonal. A p-value is calculated using Wilcoxon one-sided signed rank tests under the null hypothesis that the median performance differences between tissue-aware and tissue-agnostic Icebear model is less than or equal to 0.

bioRxiv preprint doi: https://doi.org/10.1101/2023.10.19.563173; this version posted August 12, 2024. The copyright holder for this preprint (which was not certified by peer review) is the author/funder, who has granted bioRxiv a license to display the preprint in perpetuity. It is made available under aCC-BY 4.0 International license.

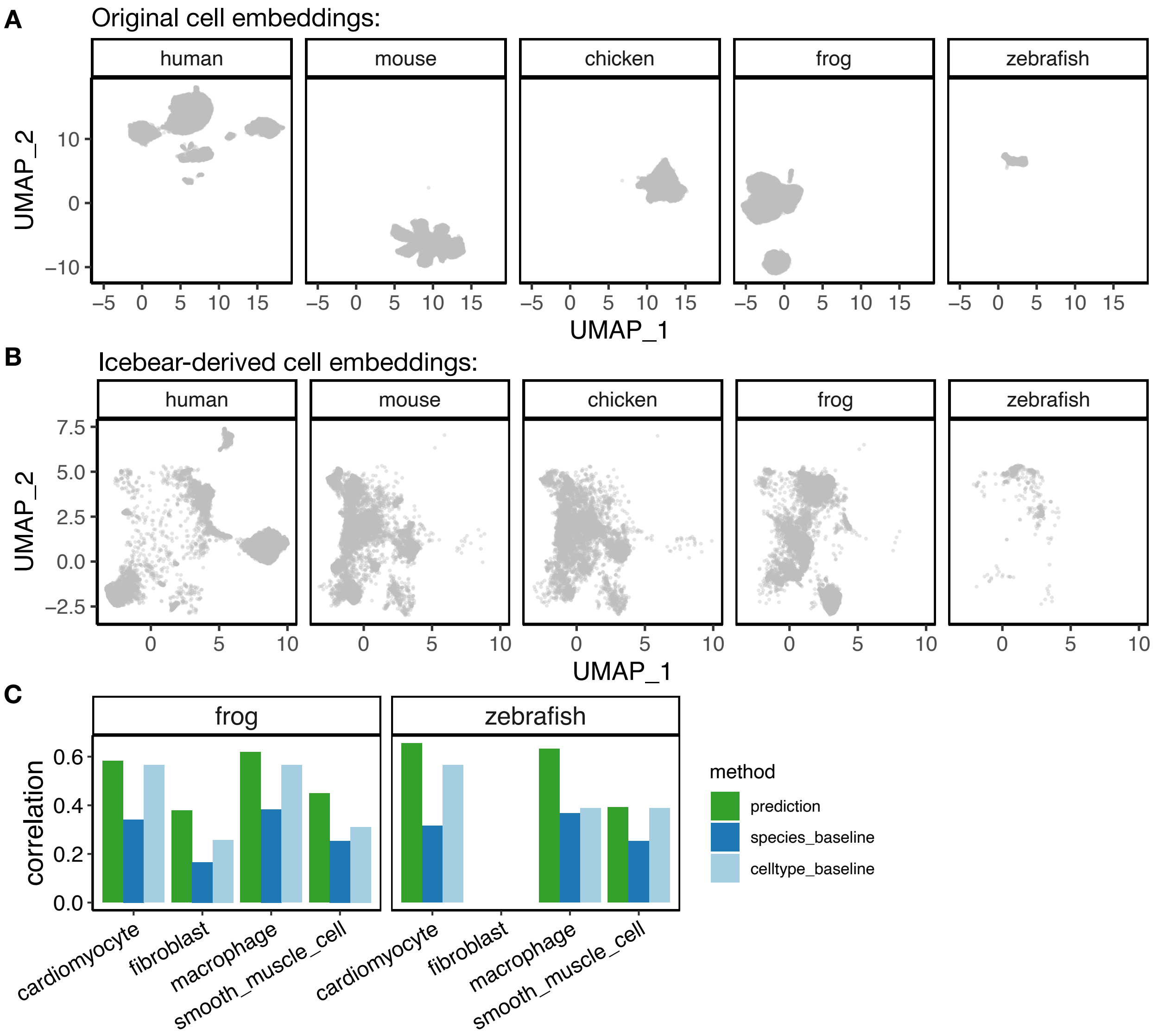

Figure S4: **Icebear can be applied to multiple species.** (A) UMAP of cell embeddings based on the original experimental measurement, cell embeddings are derived jointly and plotted separately across species. (B) UMAP of cell embeddings based on the Icebear model, cell embeddings are derived jointly and plotted separately across species. (C) Barplot showing Pearson correlation coefficient (cor) between cross-species predicted and observed gene expression profiles in the "held-out" major cell types in human heart. For each cell type, the Pearson correlation coefficient is compared between Icebear's prediction (dark green bar) and two baselines (species and celltype). Human profiles are either predicted based on cells in the frog (left) or zebrafish (right).

bioRxiv preprint doi: https://doi.org/10.1101/2023.10.19.563173; this version posted August 12, 2024. The copyright holder for this preprint (which was not certified by peer review) is the author/funder, who has granted bioRxiv a license to display the preprint in perpetuity. It is made available under aCC-BY 4.0 International license.

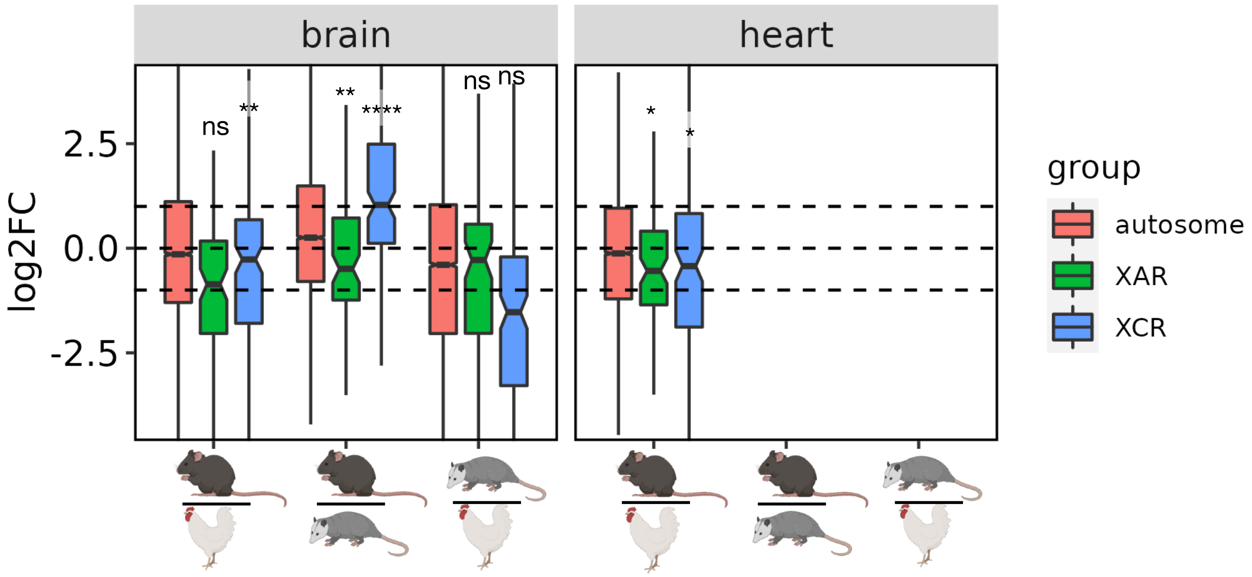

Figure S5: **Comparison of XCU patterns between Icebear and pseudobulk measurements** Boxplot of log2 fold change of pseodobulk gene expression across species, with genes grouped by their X-linked pattern. The statistical significance of XCU events is calculated using the Wilcoxon one-sample rank sum test and subjected to multiple hypothesis correction.

bioRxiv preprint doi: https://doi.org/10.1101/2023.10.19.563173; this version posted August 12, 2024. The copyright holder for this preprint (which was not certified by peer review) is the author/funder, who has granted bioRxiv a license to display the preprint in perpetuity. It is made available under aCC-BY 4.0 International license.

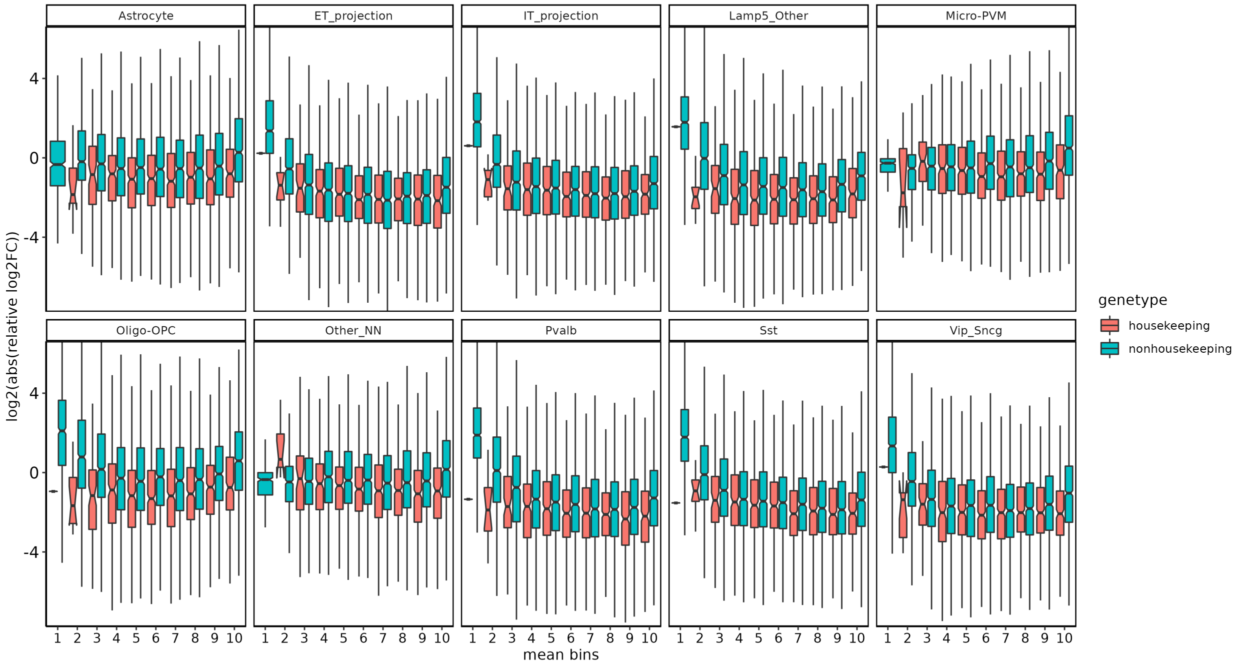

Figure S6: **Relative prediction error across cell types and levels of pseudobulk gene expression** Boxplots of genes relative prediction error across cell types. Genes are separated by their properties, and ten equal-sized expression bins are sorted from lowest (bin 1) to highest (bin 10).

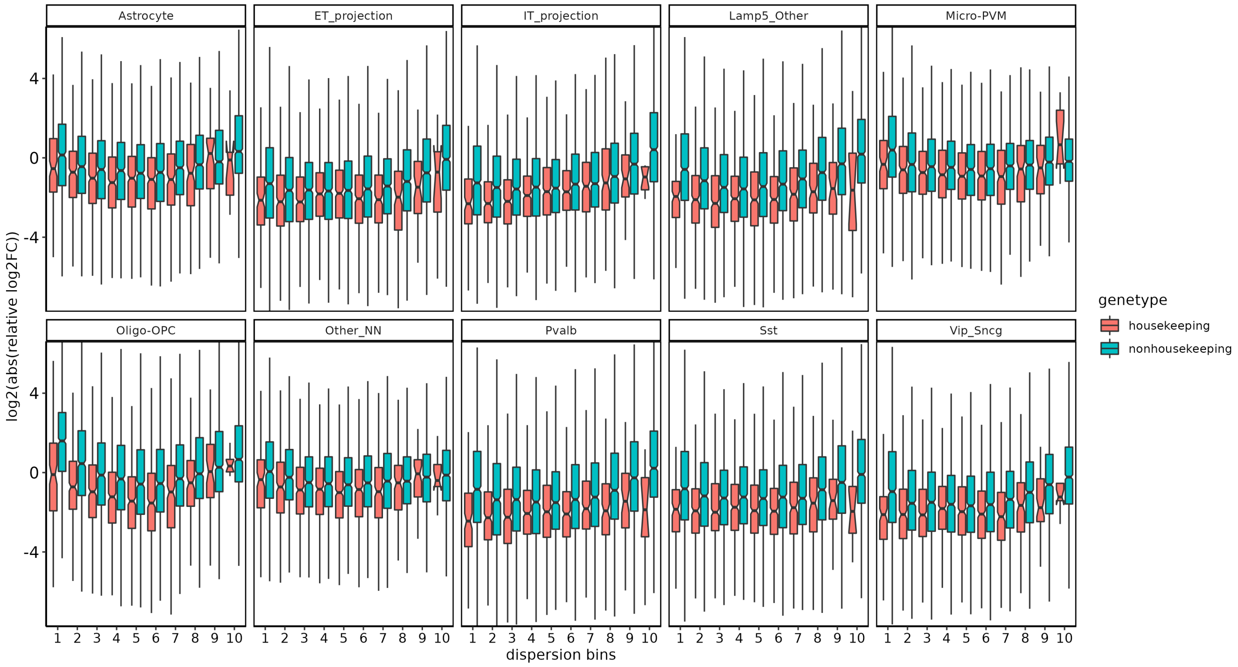

Figure S7: **Relative prediction error across cell types and levels of gene expression dispersions** Boxplots of genes relative prediction error across cell types. Genes are separated by their properties and ten equal-sized bins based on their expression dispersion across all cells, from lowest (bin 1) to highest (bin 10).

bioRxiv preprint doi: https://doi.org/10.1101/2023.10.19.563173; this version posted August 12, 2024. The copyright holder for this preprint (which was not certified by peer review) is the author/funder, who has granted bioRxiv a license to display the preprint in perpetuity. It is made available under aCC-BY 4.0 International license.

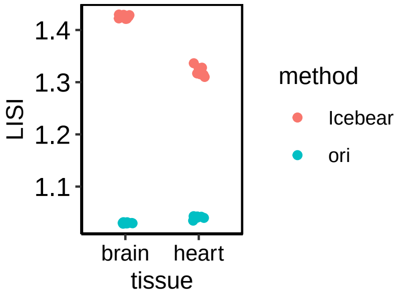

Figure S8: **Quantification of cross-species integration on the sci-RNA-seq dataset** LISI scores calculated based on Icebear's embeddings or the PCA embeddings of the original measurements. For each tissue (i.e., brain or heart), we calculate how well cells from different species are mixed together. 50,000 cells are randomly sampled from the cell population ten times, and each dot represents the LISI score on one random sampling.
